# A Few Remarks on the Fevers of Middle Tennessee and Their Treatment

**Published:** 1890-11

**Authors:** J. C. Shapard

**Affiliations:** Winchester, Tenn.


### A FEW REMARKS ON THE FEVERS OF MIDDLE TENNESSEE AND THEIR TREATMENT.

BY J. C. SHAPARD, M. D., WINCHESTER, TENN.

Read at the meeting of the Tri-State Medical Association of Alabama, Georgia and Tennessee, Oct. 15, 1890, Chattanooga, Tenn.

I have been observing the fevers of Middle Tennessee for nearly forty-five years, and studying them as best I could. I say as best I could, as my work has principally been in a small town and in the country. I have seldom had a opportunity of making post mortem examinations and therefore have not studied their lesions, neither have I been able to study their etiology with the microscope, as have some of our more fortunate brethren, but have had to content myself with giving my attention to their symptomatology, their course and the effects of treatment on them. This much said. My observations are not likely to have more importance attached to them than they deserve. And yet, I feel that my opportunities for such study have not been altogether devoid of advantages.

As is well-known, the two geological divisions of the state, the Central Basin and the Highland Rim, constitute nearly the whole of Middle Tennessee. The former is about 650 or 700 feet above the level of the sea; the latter being about 1000' above that level.

The Basin, Lower Silurian, is a very rich country and is in a very high state of cultivation. It is a limestone formation. There is lime, the carbonate, in the rocks; lime in the soil; lime in the water, and lime in everything.

The Highland Rim, 300 or 350 feet above the Basin, is in the great carboniferous formation, but is sub-divided, at least on its eastern side, into two lesser and very dissimilar divisions. The inner one, immediately surrounding the Basin, is a siliceous or sandy formation, which abounds in the best of freestone water, with many fine mineral springs, but with a very thin soil. This division being a sandy formation, water does not remain on the surface, but percolates deep down in the earth, consequently malarial fevers have always been infrequent in it.

The outer division of the Rim, sometimes called the "Red Lands," is a limestone formation, with a clay subsoil. It is a beautiful, fertile and fine farming country, but its distinctive feature, so far as this paper is concerned, is its clay subsoil, through water cannot pass, consequently it remains on the surface in ponds and marshes, and malarial fevers are, or were in early times, quite common in it. And yet there are high and dry sections of this division, where such fevers were never very prevalent. East of the Rim rise the Cumberland mountains, known to geologists as the Cumberland Table Land, which is also in the carboniferous formation and is 2000 feet above the level of the sea. It is sand-capped, with a poor soil, but its water and air are pure and it has always been almost, if not entirely exempt from malarial fevers.

This divides Middle from East Tennessee and belongs equally to both of them, at least, it will be so considered in this paper as far as Middle Tennessee is concerned.

My town in Middle Tennessee is situated in the outer division of the Rim, in the Red Lands, but near its inner division.

My work has principally been at the Rim, but I am in easy reach of the Central Basin, to which I make frequent visits, and I am also near enough to the Table Land to make an occasional visit there.

This I consider a great advantage in studying the fevers of the country, so diversified in so many respects, as varieties of soil, differences of altitude and consequent differences of temperature.

We will now take a nearer look at the fevers of Middle Tennessee.

In early times, say from the first settlement of the country up to about the year 1840, while the country was new and being cleared up and prepared for its present high state of cultivation, the fevers were altogether malarial. They were periodical, remittent and intermittent. They sometimes became congestive or malignant. They prevailed almost entirely during the summer and fall, but there was an occasional vernal return, particularly of an intermittent, and they were found along the streams and on the lowlands, the high lands being exempt from them. And I particularly call attention to the fact that they were very prevalent in the Basin, not so prevalent in the Rim, but of this division these were more prevalent in the Red Lands than on the inner or siliceous portion; in fact, they were seldom seen in that section. And they were never seen on the mountain or Table Land; at least, never originating there.

There was no difficulty in diagnosing those fevers. A differential diagnosis was hardly known then. And there was no difficulty in their treatment. Quinine cured them,and it cured them promptly. As above said, they were periodic. Quinine was an anti-periodic and enough of it given, the patient was soon well.

The fevers and most of the other diseases of that period were considered *sthenic*, and bore depletion by the lancet or otherwise to an extent that would now be considered hazardous in the extreme.

About the year 1840 a great change came over the diseases of the country—a revolution made its appearance.

Typhoid fever began its terrible career. It came as gently as the falling snow, and yet as ruthlessly as an invading army, and no pen could describe the ravages of its march. It took possession of the whole country, prevailing alike on the high lands and the low; was as prevalent, in proportion to population, on the Table Land as in the Basin, in the siliceous portion of the Rim as in the clay lands. And it had all seasons for its own. It may not have been so prevalent in summer as in winter, but it was found at all times.

It was typhoid fever everywhere and every case was typhical. And it impressed its characteristics, at least, to some extent, on most of the other diseases of the country, which from that time to this have been more or less asthenic or adynamic. No more blood-letting; harsh purgatives all laid aside, and all sedatives used with the utmost caution, and quinine ceased to be a specific.

Such and so great was the revolution. I will not say absolutely that there were not then malarial fevers to some extent along the streams and low lands of the Basin, and on the clay lands of the Rim, but if so, they were so overshadowed by the prevailing typhoid fevers as not to be observable without the most careful inquiry. But typhoid fever was prominently manifest from the highest point of the mountain to the lowest valley of the Basin. The habitat of malarial fevers was the marshes and lowlands; that of typhoid fever was the high lands, as well as the low.

Typhoid fever held absolute sway, was master of the situation about twenty years, perhaps not quite so long, when a change was again observable, but not so manifest as the one above alluded to. The symptoms of malarial fever began to be observed again, but invariably in connection with those of typhoid fever. It seemed as if, so to speak, malarial fevers were again recovering themselves, as if the conqueror had lost his prestige and the conquered were again asserting themselves. I have seen this in real life, very real, and so have my hearers. From that time the symptoms of both typhoid and malarial fevers were seen side by side in the same cases for a number of years, say fifteen or twenty. I don't know what the lesions were, neither do I know what the causes were, but the symptoms of both diseases were plainly seen by all men, even by the non-professional.

It might have been, and probably was, that the malarial symptoms were more manifest in the early stage of the disease, and the typhoid symptoms in the later stages, but the course of the disease was that of typhoid fever, was continued fever, and no amount of quinine would arrest it.

It was during this period that the theory of typho-malarial came into vogue. I hardly know how or when it happened, but I found myself, in common with most of the physicians whom I met, holding that theory. The facts that I saw all seemed to favor that theory, and I settled down in the conviction of its truth, although I had not then investigated the matter to any considerable extent.

But this theory is not incompatible with the further theory that in such a compound fever the typhoid element may predominate in one section of the country and the malarial element in another section, local causes favoring the one element in one section and the other in another, which I think was really the case. For instance, on the Table Land, where malaria is, and always was, almost unknown, the disease approaches more nearly to the old typical typhoid fever than is the case in the Basin, 1400 feet nearer the sea level, and where, in fact, it is more like the malarial fever was before the advent of typhoid fever. And this is apparent on a smaller scale. In the Barrens, or siliceous division of the Rim, it is more typhoid, while on the outer division or clay land, it is more malarial.

These two divisions of the Rim, it will be remembered, are lying side by side of each other, of the same elevation, 1000 feet above the sea, and in the same great geological formation, the carboniferous, although they differ geologically in subdivisions, the one having a sandy soil, the other a clay. This demonstrating most clearly the influence of geological formation on disease.

But, behold, another change in the fevers of the country, which has been going on for a number of years, perhaps ten or a dozen. During this period typical symptoms of both typhoid and malarial fevers have gradually disappeared, have faded away. So that we do not see them in conjunction, as was so common a few years ago. Who now ever sees a typiical case of typhoid fever, or even a typical case of malarial fever, as that disease was seen fifty years ago? Or even a typical case of typo-malarial fever as that disease was seen a few years ago? Occasionally a case of intermittent fever is seen where, in the malarial period, hundreds were seen, and if a remittent fever should make its appearance, it soon takes up the line of march as a continued fever, in combination with, or in subordination to, a typhoid fever, but not as a continued malarial fever.

I have now traced the two great fevers of Middle Tennessee, typhoid and malarial, from their origin, through their changing fortunes, in their combination and fusion down to the present time. And although the fever now, for there is only *one* fever *now*, shows none, or but few of the peculiarities of those fevers in the past. Yet, if they have not lost themselves in the fever of to-day, I don't know where they are.

I sometimes illustrate this to my satisfaction, thus: After the war our state was full of Northern and Southern people. They had their distinctive Northern and Southern peculiarties, which could be seen at a glance. But time has effaced these peculiarities, and we are now a homogeneous people—all Tennesseeans!

The sum of the whole matter is that, aside from an occasional intermittent, there is only *one fever* in Middle Tennessee to-day, which is typhoid and malarial, more typhoid in some sections and more malarial in others.

It has been maintained by some that there is a continued malarial fever *per se* in this country, which I do not believe. Malarial fevers in Middle Tennessee, so far as my observations have extended, have always been periodical, and whatever continuity there may be in them now, they owe to their union with typhoid fever, which is a continued fever.

The foregoing observations would seem to show that the fevers of Middle Tennessee have not been fixed and unchangeable in their course, but, to use a popular phrase of the day, they have been influenced by their environment. If this has been the case in the past, it will doubtless be so in the future. At least, shall believe these views to be correct until the contrary shall be proved.

However, I am well aware that the microscopists of the day are making some wonderful revelations, and I hold myself in readiness to bow to their decision whenever they show me that I am wrong.

But I am not here to-day with any iron-bound theory of fever. I mean the fever of to-day and of Middle Tennessee. I am well aware that the subject is in a somewhat chaotic condition, and I am constantly looking for new light and more light on it. I cling not to the dead past, but am ready to join hands with the living present. I am ready to abandon the terminology of the past and adopt the new definitions, if they will better,answer our purpose.

I propose to say something about the treatment of the fevers, or fever of Middle Tennessee, but it will not be much.

I don't know anything about curing a continued fever. I don't undertake to arrest it, or cut it short, as is sometimes said. And yet, I an unremitting in its management and treatment, and often see patients recover under such a course that I think would otherwise have died.

Of course, cold water has its value in the treatment of fever, but by baths I have found it impracticable in country practice, and believe it to be so everywhere outside of a hospital.

I prescribe the new anti-pyretics, particularly anti-febrine and phenacetine, and I think I accomplish much good with them, but I watch them very closely.

In old times, when malarial fevers alone prevailed, quinine cured them. They were periodic. Quinine was an anti-periodic and broke up periodic fevers, but no amount of it will arrest a continued fever for one hour. It has been claimed that in the combined fever of the country it will eliminate the malarial element, but it is not certain that it does even that much, and it is very certain that it does not arrest the disease, and, therefore, it is of questionable utility in such cases. But quinine is also an anti-pyretic, as such it is not, however, so efficient as the articles above named. And to be efficient more of it is required than is safe given throughout an attack of fever. Its anti-periodic property may make it valuable in a threatened congestion; and its tonic property may make it do good service in a convalescence.

Then, all things considered, there is, in my opinion, entirely too much quinine used in the treatment of continued fever in Middle Tennessee. More of it may, however, be profitably prescribed in some sections than in others; in the low lands than in the high lands. In excessive quantity it does great harm. It disturbs the stomach, depresses the heart and sometimes injures vision and hearing.

Physicians are often blamed nowadays for prescribing alcoholics in the treatment of disease. They are accused of making drunkards of their patients and increasing the tide of dissipation that is threatening the overthrow of our civilization. Grave charges, indeed! And the assertion is often and boldly made, that we could dispense with these destructive agents, and use other means equally valuable, but devoid of danger. In reply to all this, I only wish to say that I often think my fever patients are benefitted by the use of alcohol in some form, and that some of them, if not many, would die without it. And, as I do not know any substitute for it, I shall feel it my duty to continue to prescribe it.

